# What predicts delayed first antenatal care contact among primiparous women? Findings from a cross-sectional study in Nigeria

**DOI:** 10.1186/s12884-022-05079-y

**Published:** 2022-10-05

**Authors:** Bola Lukman Solanke, Olufemi O. Oyediran, Ayodele Aderemi Opadere, Taofik Olatunji Bankole, Olabusoye Olu Olupooye, Umar Idris Boku

**Affiliations:** 1 Department of Demography and Social Statistics, Obafemi Awolowo University, Ile-Ife, Nigeria; 2 Department of Nursing Science, Obafemi Awolowo University, Ile-Ife, Nigeria; 3 Department of Behavioral Studies, Redeemer’s University, Ede, Nigeria; 4 Department of Public Health, Osun State University, Osogbo, Nigeria; 5 Department of Sociology, Lagos State University, Ojo, Nigeria; 6 Department of Demography and Social Statistics, Federal University, Birnin-Kebbi, Nigeria

**Keywords:** Delayed first antenatal care, Primiparous women, Maternal and newborn health, Nigeria

## Abstract

**Background:**

Delayed first antenatal care contact refers to first antenatal care contact occurring above twelfth weeks of gestation. Studies in Nigeria and in other countries have examined the prevalence and predictors of delayed first antenatal care contact. Nevertheless, existing studies have rarely examined the predictors among primiparous women. In addition, the evidence of higher health risks associated with primigravida emphasizes the need to focus on primiparous women. This study, therefore, examined the predictors of delayed first antenatal care contact among primiparous women in Nigeria.

**Methods:**

The study was a descriptive cross-sectional design that analyzed data extracted from the 2018 Nigeria Demographic and Health Survey. The study analyzed a weighted sample of 3,523 primiparous women. The outcome variable was delayed first antenatal care contact. explanatory variables were grouped into predisposing, enabling, and need factors. The predisposing factors were maternal age, education, media exposure, religion, household size, The knowledge of the fertile period, and women’s autonomy. The enabling factors were household wealth, employment status, health insurance, partner’s education, financial inclusion, and barriers to accessing healthcare. The need factors were pregnancy wantedness and spousal violence during pregnancy. Data were analyzed using Stata 14. Two multivariable logistic regression models were fitted. Statistical significance was set at p < 0.05.

**Results:**

Nearly two-thirds (65.0%) of primiparous women delayed first antenatal care contact. Maternal age, maternal education, media exposure, religion, household membership, and knowledge of the fertile period were predisposing factors that significantly influenced the likelihood of delayed first antenatal care contact. Also, household wealth, employment status, health insurance, partner’s education, perception of distance to the health facility, and financial inclusion were enabling factors that had significant effects on delayed first antenatal care contact. Pregnancy wantedness was the only need factor that significantly influenced the likelihood of delayed first antenatal care contact.

**Conclusion:**

The majority of primiparous women in Nigeria delayed first antenatal care contact and the delay was predicted by varied predisposing, enabling, and need factors. Therefore, a public health education program that targets women of reproductive age especially primiparous women is needed to enhance early antenatal care contact in the country.

## Background

Delayed first antenatal care contact (also known as late antenatal booking) refers to first antenatal care contact occurring after twelfth or more weeks of gestation [1]. The practice of delaying first antenatal care contact undermines the successful implementation of current global model of standard antenatal care being implemented in Nigeria [1, 2]. The model recommended that pregnant women should make the first antenatal care contact with skilled health personnel early, preferably between eight and twelfth weeks of gestation [1]. The essence of early first antenatal care contact is to avail skilled health providers the opportunity for prompt diagnosis, management of infections, and early detection of other potential obstetric complications, and to prescribe appropriate medication and counseling to pregnant women [3]. This is achieved through antenatal care contacts where pregnant women are educated on appropriate health-seeking behaviors during pregnancy, pregnancy danger signs, and vital family planning methods [4]. Additional evidence across the world suggests that early initiation of antenatal care contact promotes the use of skilled birth attendants [5], enhances institutional delivery and early postnatal check [6, 7], reduces the risks of miscarriage [8], encourages women to attain the recommended eight antenatal care contacts [1], and also to improve the general state of maternal and newborn health.

In spite of this evidence, many pregnant women particularly in developing countries still delay first antenatal care contact due to diverse reasons such as the absence of pregnancy signs or sickness [9], long waiting time at a health facility [10], socio-cultural barriers to health services [11], poor perception of pregnancy complications [12, 13], prior pregnancy experience [14], and some other health service challenges [15, 16]. The consequences of delayed first antenatal care contact for maternal and newborn health make it an important public health concern in many developing countries. This not only underscores the need for further research to unravel its prevalence and predictors, but also for more research to provide additional policy-relevant information. Studies in Nigeria [17–20] and other countries have examined the prevalence and predictors of delayed first antenatal care contact [21–27]. Many of these studies [21, 25] found prevalence as high as 75–85% in different developing countries, which further justifies continued research attention on the underlying factors of delayed first antenatal care contact.

Nevertheless, existing studies have rarely examined the predictors among primiparous women (first-time mothers), particularly in Nigeria. Though a recent study in Ghana [28] focused on first-time mothers, the attention of the study was not strictly on delayed first antenatal care contact. Besides, the data analyzed in the study was not nationally representative but extracted from a Health and Demographic Surveillance System (HDSS). This may undermine inferences from the study. The paucity of studies on primiparous women hinders a nuanced understanding of antenatal care contacts among first-time mothers in many countries. Primiparous women are a peculiar segment of childbearing women who needs to be well-mobilized and guided in all aspects of motherhood. Though the transition to motherhood is usually characterized by positive emotions and expectations [29], the period is, however, replete with a lot of personal difficulties and systemic challenges [30] that could affect how first-time mothers respond to issues within the continuum of maternity care.

In a recent study conducted in Nigeria among first-time young parents, it was found that a number of social and health system factors promoted the use of maternal health services including antenatal care. These included the availability of high-quality care, positive health-seeking behavior, equitable gender norms, social support for primiparous women, and social and economic power of first-time parents. The study further observed some social and systemic barriers to an adequate use of maternal healthcare services among first-time parents. Such barriers included inadequate birth preparedness, stigmatization of adolescent pregnancy and motherhood, religious barriers, poor health habits, and gender inequality. Though the study targeted only first-time parents in six states of Nigeria, its call for existing strategies to transform social norms for the benefit of maternal and newborn health reinforces calls that first-time parents including first-time fathers [31] be specially targeted for an in-depth understanding of their peculiarities as well as the provision of appropriate guidance to them [32]. Also, the evidence of elevated obstetric risk of primiparity such as low birth weight, emergency cesarean section, and prolonged labor [33–35] emphasizes the need to focus on primiparous women.

Hence, this study examined the predictors of delayed first antenatal care contact among primiparous women in Nigeria. Findings from the study will provide more inputs for the 2021 revised national population policy which seeks among other targets to increase the antenatal care attendance rates from the current 67–87% at the end of 2030 [4, 36]. The study was guided by the research question: what predicts delayed first antenatal care contact among primiparous women? The Andersen behavioral model of health services use [37, 38] provided the theoretical underpinning of the study. The model asserts that the utilization of health services is influenced mainly by three types of factors, namely, predisposing factors (the socio-demographic characteristics of individuals that may serve either as a facilitator or barrier to healthcare use prior to the need for the service), enabling factors (the individual, family or health service factors that enhance access to specific health service), and need factors (the conditions that show the potential need for specific health service). A number of studies [39–41] have elucidated antenatal care contacts using the Andersen behavioral model.

## Methods

### Study design

The study was descriptive cross-sectional research that entails the analysis of quantitative secondary data extracted from the women’s data of 2018 Nigeria Demographic and Health Survey (NDHS). The choice of the 2018 NDHS stems from the high quality of the data, as well as the availability of the datasets in the public domain, which encourages replication of the study elsewhere, as well as the international comparability of the study findings.

### Data source

The study represents a further analysis of the 2018 NDHS, which is conducted under the auspices of the Demographic and Health Survey (DHS) program. The DHS program is being implemented in several developing countries by the Inner-City Fund (ICF) to build capacity for the collection and provision of reliable national estimates of demographic and health characteristics in developing countries [42]. The 2018 NDHS was conducted by the National Population Commission (NPC) with the technical, and financial support of many development partners [4]. The methodology adopted for the conduct of the 2018 NDHS is similar to the methodology usually adopted in the DHS program [43]. Details of the methodology are widely available to all interested researchers via https://dhsprogram.com/pubs/pdf/FR359/FR359.pdf.

### Population and sample

The study targets reproductive-age women who are first-time mothers in Nigeria. In the 2018 NDHS, this group of childbearing women was 11,363 (27.2%) out of the 41,821 women covered in the survey but only 3,488 of the women were included in the domestic violence module. The study sample was derived upon execution of the inclusion/exclusion criteria. Three sets of women were excluded. One, all women who were not first-time mothers were not included. This was necessary to maintain study focus on only primiparous women. This criterion excluded 30,458 women covered in the survey. Two, all women not included in the domestic violence module were excluded. This was necessary because only women included in the module were asked questions on spousal violence, which is one of the explanatory variables examined in the study. This criterion excluded 7,875 women covered in the survey. Three, 24 women who reported traditional or other religions besides Islam and Christianity were excluded due to their insignificant proportion which may distort the statistical analysis. The analyzed sample in the study was therefore 3,523 women. The DHS weighting factor was applied.

### Outcome variable

The outcome variable in the study was delayed first antenatal care contact. This was derived from response to the timing of the first antenatal care contact. All first antenatal contact occurring after 12 weeks of gestation were grouped as ‘yes’ and coded ‘1’ while contact within the first trimester was grouped as ‘no’ and coded ‘0’. This measure is in line with the recommendation of the current global model of standard antenatal care [1] and has been adopted in existing studies on delayed first antenatal care contact [18, 23, 24, 26, 27].

### Explanatory variables

Findings in existing empirical studies, as well as the Andersen model, guided the selection of the explanatory variables. Four sets of variables were selected. One, seven predisposing factors were selected. These were maternal age (15–24, 25–34, or 35–49 years), education (no formal education, primary, secondary, or higher), media exposure (low, moderate, or high), religion (Islam or Christianity), household size (small or large, with small size indicating six people, and large size indicating seven or more people in the household), knowledge of fertile period (correct or incorrect, with correct knowledge indicating midway between two menstrual cycles) and women’s autonomy (low or high, with low indicating women’s lack of involvement in the household decision, and high indicating sole or joint involvement with a partner). Media exposure was derived by combining responses to the frequency of reading newspaper, listening to radio, and watching television per week. Responses to each media outlets were assigned score of ‘1’ for ‘not at all’ ‘2’ for ‘less than once a week’ ‘3’ for ‘at least once a week’. This gives a total score of nine (9) which was divided into three equal parts with scores of ‘1–3’ representing ‘low exposure’, scores of ‘4–6’ representing ‘moderate exposure’, and scores of ‘7–9’ representing ‘high exposure’.

Household size was divided into ‘small or large’ based on the recommendation of the 1988 national policy on population for development, unity, progress, and self-reliance [44] which suggests four children per woman. A large household size connotes that the household consists of more than four other people in addition to the couple. The knowledge of the fertile period was included because late awareness that pregnancy has occurred contributes to delayed first antenatal care [13]. Women’s autonomy was derived by combining participation in the three-household decision-making. Women who had the final say either solely or jointly with their partner in all the decisions were deemed to have ‘high autonomy’ while other women belong to the ‘low autonomy’ category. These variables have been found to be important correlates of delayed first antenatal care in existing studies [18, 25, 28, 45]. Two, six enabling factors were selected. These were household wealth (poorest, poorer, middle, richer, richest), employment status (employed or unemployed), health insurance (enrolled or not enrolled), partners’ education (none, primary, secondary, higher), financial inclusion (yes or no, with yes indicating ownership of a bank account and no indicating otherwise), and perception of money for medical treatment and perception of distance to health facility (big problem or not a big problem). Bank ownership was used to measure women’s financial inclusion because it is one of the key means that ensures access to formal financial information, assistance, and services such as credit and insurance. This measure is widely accepted and used to proxy financial inclusion [46, 47].

With the exclusion of financial inclusion, these variables have been confirmed to be significant predictors of delayed first antenatal care contact [18, 23, 24, 45, 48, 49]. Three, two need factors were selected. These were pregnancy wantedness (planned, when pregnancy was wanted at the time of occurrence or unplanned when pregnancy was not wanted at all or not wanted at the time of occurrence) and spousal violence during pregnancy (experienced or not experienced). The two variables have been identified as having significant predictive power on antenatal care utilization [49–52]. Two external environmental factors, namely, place of residence and geo-political zone were included for statistical control. Studies have shown that these two variables are strong correlates of delayed first antenatal care contact [26, 53].

### Data analysis

Data were analyzed at three levels using Stata 14 [54]. Firstly, the prevalence of delayed first antenatal care contact and the socio-demographic characteristics of respondents were described using frequency distribution and percentages. Secondly, the research variables were cross-tabulated for the purpose of assessing how delayed the first antenatal care contact varies in response to changes in the explanatory variables. The Unadjusted Odds Ratio (UOR) was used to examine the relationship between the outcome and explanatory variables. Any variable with no statistical significance at this level was not included in subsequent analysis. Thirdly, two multivariable logistic regression models were fitted to examine the predictors of delayed first antenatal care contact using Adjusted Odds Ratio (AOR). Model 1 included the predisposing, enabling, and need factors, while Model 2 controlled for the external environmental factors. Model 2 is the full model on which the discussion of findings was anchored. Statistical significance was set at p < 0.05.

## Results

### Univariate results

Table 1 presents the socio-demographic profile of the respondents. Nearly two-thirds (65.0%) of primiparous women delayed first antenatal care contact. Primiparous women in the age group of 25–34 years compared to other age groups were dominant in the sample (42.1%). More than two-fifths (42.6%) of the primiparous women attained secondary education, while nearly one-third (31.5%) of them had no formal education. More than half (58.5%) of the primiparous women had a high level of autonomy in household decisions. Primiparous women who had moderate media exposure were dominant (46.7%) in the sample compared to other women. Religious affiliation was nearly equal among the women, Christian primiparous women were however slightly more than Muslim primiparous women in the sample (51.5% vs. 48.5%). The majority (74.7%) of the primiparous women had a small household size. Likewise, the majority (75.2%) of the primiparous women had incorrect knowledge of the fertile period.

**Table 1:** Prevalence of delayed first antenatal care contact and Socio-demographic characteristics of respondents

Characteristic	Number of women	Frequency (%)	Characteristic	Number of women	Frequency (%)
Delayed first antenatal care contact			Employment status		
No	1,233	35.0	Unemployed	991	28.1
Yes	2,290	65.0	Employed	2,532	71.9
Maternal age (years)			Health insurance		
15–24	889	25.2	Not enroll	3,422	97.2
25–34	1,481	42.1	Enrolled	101	2.8
35–49	1,153	32.7	Partners’ education		
Maternal education			None	1,176	33.4
None	1,109	31.5	Primary	424	12.0
Primary	517	14.7	Secondary	1,340	38.0
Secondary	1,500	42.6	Higher	483	16.6
Higher	397	11.2	Perception of money for medical treatment		
Women’s autonomy			Big problem	1,640	46.5
Low	1,460	41.5	Not a big problem	1,883	53.5
High	2,063	58.5	Perception of distance to health facility		
Media exposure			Big problem	973	27.6
Low	995	28.5	Not a big problem	2,550	72.4
Moderate	1,645	46.7	Financial inclusion		
High	883	25.1	No	2,601	73.8
Religion			Yes	922	26.2
Christianity	1,814	51.5	Pregnancy wantedness		
Islam	1,709	48.5	Planned	3,061	97.2
Household size			Unplanned	462	2.8
Small	2,630	74.7	Spousal violence during pregnancy		
Large	893	25.3	Not experienced	3,424	97.2
Knowledge of fertile period			Experienced	99	2.8
Correct	873	24.8	Place of residence		
Incorrect	2,650	75.2	Urban	1,730	49.1
Household wealth quintile			Rural	1,793	50.9
Poorest	537	15.3	Geo-political zone		
Poorer	656	18.6	North-Central	481	13.7
Middle	718	20.4	North-East	481	13.7
Richer	801	22.7	North-West	829	23.5
Richest	811	23.0	South-East	388	11.0
			South-South	412	11.7
			South-West	932	26.5
Total	3,523	100.0	Total	3,523	100.0

Household wealth was progressive among the respondents as the proportion of primiparous women in each household wealth group increased consistently from the poorest to the richest households. The majority (71.9%) of the primiparous women were employed. Virtually all the primiparous women were not enrolled in any health insurance scheme. Slightly more than one-third of the respondents’ partners had no formal education. However, secondary education was the dominant level attained among those who had formal education. Nearly half (46.5%) of the primiparous women perceived money for medical treatment as a ‘big problem’ while slightly more than a quarter (27.6%) of them perceived distance to health facilities as a ‘big problem’. The majority of the primiparous women (73.8%) did not own a bank account. The majority (86.9%) of the primiparous women reported that their pregnancies were planned. Less than 5% (2.8%) of the primiparous women reported experiencing spousal violence during pregnancy. Almost equal proportions of the primiparous women reside either in the urban or rural areas (49.1% vs. 50.9%). Primiparous women from the southwest (26.5%) and northwest (23.5%) geo-political zones were dominant in the sample.

### Bivariate results

Table 2 presents the prevalence of delayed first antenatal care contact by socio-demographic characteristics of primiparous women. The table also provides information on the statistical significance of the relationship between delayed first antenatal care contact and each of the explanatory variables. Delayed antenatal care contact was higher among younger primiparous women compared to older primiparous women. Delayed first antenatal care contact consistently declined as educational attainment improved among the primiparous women. A higher level of delayed first antenatal care contact was observed among primiparous women with high autonomy compared to those with low autonomy (81.2% vs. 67.9%). Improvement in media exposure occasioned a consistent reduction in the level of delayed first antenatal care contact. The prevalence of delayed first antenatal care contact was higher among Muslim primiparous women compared to Christian primiparous women (84.5% vs. 67.3%). Likewise, a higher prevalence of delayed first antenatal care contact was observed among primiparous women who reside in households with large sizes compared to those in households with small sizes (84.0% vs. 72.8%). Primiparous women who had incorrect knowledge of the fertile period reported slightly higher levels of delayed first antenatal care contact compared to those who had correct knowledge (76.6% vs. 72.8%).

**Table 2:** Prevalence (%) of delayed first antenatal care contact by socio-demographic characteristics

Characteristic	Prevalence	UOR	95% CI	Characteristic	Prevalence	UOR	95% CI
Maternal age				Health insurance			
15–24 ^RC^	80.5	-	-	Not enrolled ^RC^	76.4	-	-
25–34	72.5	0.636**	0.497–0.814	Enrolled	51.1	0.323**	0.193–0.540
35–49	76.0	0.765*	0.591–0.989	Partners’ education			
Maternal education				None ^RC^	86.6	-	-
None ^RC^	88.8	-	-	Primary	74.2	0.442**	0.321–0.608
Primary	78.8	0.469**	0.341–0.644	Secondary	69.1	0.344**	0.271–0.436
Secondary	68.8	0.278**	0.214–0.360	Higher	69.7	0.355**	0.259–0.487
Higher	60.6	0.194**	0.136–0.277	Perception of money for medical treatment			
Women’s autonomy				Big problem ^RC^	78.2	-	-
Low ^RC^	67.9	-	-	Not a problem	73.5	0.774*	0.624–0.961
High	81.2	2.043**	1.697–2.460	Perception of distance to health facility			
Media exposure				Big problem ^RC^	81.6	-	-
Low ^RC^	86.0	-	-	Not a problem	73.4	0.624*	0.478–0.814
Moderate	75.3	0.494**	0.387–0.631	Financial inclusion			
High	64.7	0.298**	0.229–0.389	No ^RC^	81.9	-	-
Religion				Yes	58.2	0.308**	0.251–0.378
Christianity ^RC^	67.3	-	-	Pregnancy wantedness			
Islam	84.5	2.649**	2.121–3.308	Planned ^RC^	75.5	-	-
Household size				Unplanned	76.9	1.250**	1.109–1.392
Small ^RC^	72.8	-	-	Spousal violence during pregnancy			
Large	84.0	1.969**	1.541–2.516	Not experienced ^RC^	75.6	-	-
Knowledge of fertile period				Experienced	75.5	0.991	0.547–1.794
Correct ^RC^	72.8	-	-	Place of residence			
Incorrect	76.6	1.302**	1.109–1.495	Urban ^RC^	69.7	-	-
Household wealth quintile				Rural	81.4	1.899**	1.529–2.361
Poorest ^RC^	91.8	-	-	Geo-political zone			
Poorer	82.4	0.417**	0.282–0.616	North-Central ^RC^	74.6	-	-
Middle	78.4	0.323**	0.222–0.471	North-East	84.5	1.865**	1.324–2.627
Richer	73.3	0.244**	0.167–0.358	North-West	91.0	3.428**	2.342–5.018
Richest	59.3	0.129**	0.089–0.189	South-East	67.6	0.710	0.504–1.002
Employment status				South-South	71.2	0.839	0.607–1.160
Unemployed ^RC^	83.9	-	-	South-West	63.3	0.586*	0.426–0.807
Employed	72.5	0.506**	0.403–0.636				

Notes: RC (Reference category), *p < 0.05 (relationship significant), **p < 0.01 (relationship significant)

As household wealth improved, the level of delayed first antenatal care contact declined consistently. Unemployed primiparous women had higher prevalence of delayed first antenatal care contact compared to employed primiparous women (83.9% vs. 72.5). Similarly, while the prevalence of delayed first antenatal care contact was 76.4% among the larger group of women who enrolled in health insurance, the prevalence was lower (51.1%) among the smaller proportion of primiparous women enrolled in health insurance. Except at higher education levels, delayed first antenatal care contact declines consistently as partners’ education improves. Primiparous women who did not perceive either money for medical treatment or distance to a health facility as a big problem reported lower levels of delayed first antenatal care contact compared to other primiparous women. Also, a lower level of delayed first antenatal care contact was observed among financially included primiparous women. A slightly higher prevalence of delayed first antenatal care contact was observed among primiparous women whose pregnancies were unplanned compared to those whose pregnancies were planned (76.9% vs. 75.5%). No serious change in the level of delayed first antenatal care contact was observed among primiparous women who either experienced or do not experience spousal violence during pregnancy. Primiparous women in rural areas, as well as those in the northeast and northwest zones of the country, had higher levels of delayed first antenatal care contact. The result of the unadjusted odds ratios as shown in Table 2 reveals that with the exclusion of spousal violence during pregnancy, all the explanatory variables were significantly related to delayed first antenatal care contact.

### Multivariable results

Table 3 presents the effects of predisposing, enabling, and need factors on the likelihood of delayed first antenatal care contact among primiparous women. Four variables, namely, women’s autonomy, media exposure, perception of money for medical treatment, and perception of distance to health facility as barriers revealed no significant influence on delayed first antenatal care contact in Model (1) Also in the model, the maternal age group of 35–49 showed no statistical significance. However, media exposure and perception of distance to health facilities as barriers were strengthened in Model (2) In Model 2, six predisposing factors, namely, maternal age, maternal education, media exposure, religion, household size, and knowledge of fertile period significantly influenced the likelihood of delayed first antenatal care contact. Older and more educated primiparous women had lower odds of delayed first antenatal care contact compared to younger and uneducated primiparous women. Primiparous women who had high media exposure were less likely to delay first antenatal care contact compared to those who had low media exposure (AOR = 0.881, 95% CI: 0.477–0.814). Muslim primiparous women were more likely to delay first antenatal care contact compared to Christian primiparous women (AOR = 1.400, 95% CI: 1.070–1.832). Primiparous women in households with large membership had higher odds of delayed first antenatal care contact compared to those in households with small membership (AOR = 1.376, 95% CI: 1.092–1.733). Likewise, primiparous women who had incorrect knowledge of the fertile period were more than twice more likely to delay first antenatal care contact compared to those who had correct knowledge (AOR = 2.942, 95% CI: 2.358–3.671).

**Table 3:** Effects of predisposing, enabling and need factors on delayed first antenatal care contact

Characteristic predicting delayed first antenatal care contact	Model 1		Model 2	
	AOR	95% CI	AOR	95% CI
Maternal age				
15–24 ^RC^	1.000	-	1.000	-
25–34	0.594**	0.461–0.728	0.470*	0.021–0.919
35–49	0.949	0.445–1.452	0.811**	0.675–0.946
Maternal education				
None ^RC^	1.000	-	1.000	-
Primary	0.469**	0.341–0.644	0.922	0.554–1.531
Secondary	0.278**	0.214–0.360	0.494**	0.387–0.631
Higher	0.193**	0.136–0.277	0.298**	0.229–0.389
Women’s autonomy				
Low ^RC^	1.000	-	1.000	-
High	1.123	0.900–1.400	1.126	0.900-1.408
Media exposure				
Low ^RC^	1.000	-	1.000	-
Moderate	1.008	0.771–1.320	1.042	0.789–1.378
High	0.881	0.637–1.219	0.623*	0.477–0.814
Religion				
Christianity ^RC^	1.000	-	1.000	-
Islam	1.505*	1.167–1.942	1.400*	1.070–1.832
Household size				
Small ^RC^	1.000	-	1.000	-
Large	1.392*	1.071–1.809	1.376*	1.092–1.733
Knowledge of fertile period				
Correct ^RC^	1.000	-	1.000	-
Incorrect	2.043**	1.697–2.460	2.942**	2.358–3.671
Household wealth quintile				
Poorest ^RC^	1.000	-	1.000	-
Poorer	0.525*	0.351–0.787	0.535*	0.355–0.804
Middle	0.571*	0.382–0.855	0.585*	0.391–0.879
Richer	0.528*	0.340–0.819	0.551*	0.352–0.860
Richest	0.388**	0.238–0.631	0.424*	0.258–0.697
Employment status				
Unemployed ^RC^	1.000	-	1.000	-
Employed	0.703*	0.547–0.904	0.774*	0.624–0.961
Health insurance				
Not enrolled ^RC^	1.000	-	1.000	-
Enrolled	0.443*	0.248–0.793	0.371*	0.207–0.670
Partners’ education				
None ^RC^	1.000	-	1.000	-
Primary	0.588*	0.416–0.832	0.578*	0.408–0.819
Secondary	0.726*	0.545–0.967	0.719*	0.540–0.957
Higher	1.181	0.789–1.968	1.214	0.798–1.848
Perception of money for medical treatment				
Big problem ^RC^	1.000	-	1.000	-
Not a problem	1.130	0.893–1.429	1.133	0.893–1.438
Perception of distance to health facility				
Big problem ^RC^	1.000	-	1.000	-
Not a problem	0.803	0.606–1.062	0.725*	0.550–0.956
Financial inclusion				
No ^RC^	1.000	-	1.000	-
Yes	0.523**	0.404–0.677	0.537**	0.414–0.695
Pregnancy wantedness				
Planned ^RC^	1.000	-	1.000	-
Unplanned	1.574**	1.460–1.697	1.210*	1.075–1.361
Place of residence				
Urban ^RC^			1.000	-
Rural			0.862	0.670–1.110
Geo-political zone				
North-Central ^RC^			1.000	-
North-East			1.237	0.852–1.796
North-West			2.248**	1.481–3.41
South-East			1.179	0.779–1.785
South-South			1.306	0.920–1.854
South-West			0.817	0.586–1.140

Notes: *p < 0.05, **p < 0.01

Six enabling factors, namely, household wealth, employment status, health insurance, partners’ education, perception of distance to a health facility, and financial inclusion had significant effects on delayed first antenatal care contact. Compared to primiparous women in the poorest households, the odds of delayed first antenatal care contact were lower among primiparous women in other household wealth groups. Employed primiparous women were less likely to delay first antenatal care compared to unemployed primiparous women (AOR = 0.774, 95% CI: 0.624–0.961). Also, primiparous women who enrolled in health insurance were less likely to delay first antenatal care contact compared to those not enrolled (AOR = 0.371, 95% CI: 0.207–0.670). Partners’ primary (AOR = 0.578, 95% CI: 0.408–0.819) and secondary (AOR = 0.719, 95% CI: 0.540–0.957) education resulted into less likelihood of delayed first antenatal care contact. Likewise, the odds of delayed first antenatal care contact were lower among primiparous women who did not perceive the distance to a health facility as a big problem (AOR = 0.725, 95% CI: 0.550–0.956), as well as among the financially included primiparous women (AOR = 0.537, 95% CI: 0.414–0.695). Pregnancy wantedness was the only need factor that influenced the likelihood of delayed first antenatal care contact. Primiparous women whose pregnancies were unplanned were more likely to delay first antenatal care contact compared to those whose pregnancies were planned (AOR = 1.210, 95% CI: 1.075–1.361). The odds of delayed first antenatal care contact were higher among primiparous women in the northwest zone of the country compared to primiparous women in the reference category (AOR = 2.248, 95% CI: 1.481–3.413).

## Discussion

This study assessed the predictors of delayed first antenatal care contact among primiparous women in Nigeria. The study found a high level (65.0%) of delayed first antenatal care contact among primiparous women in Nigeria. This prevalence is lower than the 74% reported in a Nigerian study where 62% of first antenatal care contact was initiated in the second trimester and 12% initiated in the third trimester [18]. Also, the finding could be compared to the 64% reported in an earlier Ethiopian study [45]. It is, however, lower than the higher figures of 75%, and 71.2% respectively reported in two recent studies in Cameroon and Ethiopia [25, 26]. The implication of the finding in the current study is that the majority of primiparous women in Nigeria do not have their first antenatal care contact in the first trimester. This is not substantially different from what the 2018 NDHS reported about antenatal care visits among the general population of pregnant women in the country [4] and seems to suggest that late initiation of first antenatal care contact is a regular feature of childbearing women in the country. Additional steps are thus needed in the country to address all known reasons for delayed first antenatal care contact among pregnant women in the country.

Existing studies have already observed that many pregnant women have a poor perception of early antenatal care contact. Some believed that pregnancy is not different from other health conditions [12], and therefore not deserving of any form of urgency. Some others believed that in the absence of visible danger signs of pregnancy or pregnancy-related sickness [9], early antenatal care contact is not compelling. There are also pregnant women who believed that pregnancy at the early stage should be kept secret to avoid miscarriage [14]. Such women thus, wrongly perceived that early initiation of antenatal care makes the pregnancy vulnerable to adverse outcomes. Studies also suggest that though some pregnant women have good knowledge of the importance of early initiation of antenatal care contact, they may however fail to initiate early contact due to a series of constraints [11, 15] such as the need for additional cost, cultural norms, poor local healthcare delivery system, or religious practices including Muslim women preference for female healthcare personnel. It is therefore crucial that more maternal healthcare strategies should be devised not only to capture these constraints but also to correct existing misinformation and misconception about the early initiation of antenatal care contact through public health education programs that target different groups of women, especially primiparous women. The development and execution of such a program will enhance the possibility of achieving the current target of increasing antenatal care attendance to 87% by 2030 [36].

The study also reveals that the socio-demographic characteristics of primiparous women in Nigeria predispose them to delayed first antenatal care contact. As found in the study and in agreement with existing studies [26, 49], older primiparous women were less likely to delay first antenatal care contact compared to mothers aged 15–24 years. One reason that may account for this finding is that younger primiparous women may not initiate antenatal contact promptly if the health facility in the community is not youth-friendly. A number of existing Nigerian studies [55, 56] have provided evidence that the respect and dignity accorded to pregnant and nursing mothers, especially in public health facilities leaves much to be desired. This may discourage some pregnant mothers from initiating early antenatal care booking. On the other hand, older primiparous women may not delay their first antenatal contact because some of them take their first pregnancy as precious, and they will want to get the best care to preserve the pregnancy and deliver safely. This is more likely to happen when pregnant women reside in households of large size.

The study further found that educated primiparous women were not likely to delay first antenatal care contact. This is consistent with earlier findings [18, 26, 45, 53] and implies that initiatives seeking to improve antenatal care coverage should continue to leverage the important role education is playing in improving the health-seeking behavior of women. Besides the education provided during antenatal care attendance, more educational messages could be spread through the mass media, which was also found to be a significant predictor in the study. The study also found that correct knowledge of the fertile period also predicted delayed first antenatal care contact. It is possible that the reason some first-time mothers failed to initiate early antenatal care contact is the late realization that they are pregnant. As observed in two studies from Ghana and Tanzania [9, 13], some pregnant women did not initiate antenatal care booking because they did not see visible signs of pregnancy. It is thus important to educate more women on how to identify the fertile period as well as the signs that may manifest after conception.

Another important finding of the study was that the enabling factors (employment, financial inclusion, partners’ education, household wealth, health insurance, and perception of distance to health facilities) lowered the odds of delayed first antenatal care contact among primiparous women. These agree with earlier findings [18, 23, 24, 49]. The enabling factors either empowers primiparous women to afford healthcare cost including the cost of antenatal care if it is not free or provides them with proper education and appreciation of antenatal care, which often leads to prompt seeking of antenatal care. Thus, maternal healthcare strategies in the country should on a continuous basis assess factors that facilitate the early initiation of antenatal care among different categories of women. This may provide the information needed to improve antenatal care program design in the country. Finally, the study reveals that pregnancy wantedness drives delayed first antenatal care contact. As observed in some existing studies [23, 26, 49, 53], women who had unplanned pregnancies often initiate antenatal care late. It is plausible to argue that many women with unplanned pregnancies may first consider terminating the pregnancy, which discourages them from booking antenatal care.

### Strengths and limitations

The basic strength of the study is the focus on primiparous women, which represents a knowledge gap rarely filled by existing studies. The analysis of nationally representative data further strengthened the international comparability of the study design, execution, and findings. The analyzed data are available and accessible to any interested person for verification of the study findings. By providing empirical support for the Andersen behavioral model of health services use, the study shed light on the applicability of the model. Notwithstanding, the findings in the study are limited only to first-time mothers. Some of the results may not resonate with multiparous or grand multiparous women. This is because a number of variables were excluded from the analysis. Also, the study makes no pretense to establish a cause-effect relationship between the research variables. This is because the data analyzed was cross-sectional in nature.

## Conclusion

The study examined the predictors of delayed first antenatal care contact among primiparous women in Nigeria. A high level of delayed first antenatal care contact exists among primiparous women in Nigeria due to varied predisposing, enabling, and need factors. A public health education program as well as financial empowerment schemes that target different groups of women, especially primiparous women are needed in the country to boost early antenatal care contact.

## Data Availability

Data analyzed in the study is available in the public domain. Interested persons or researchers could access it online at. https://dhsprogram.com/data/dataset/Nigeria_Standard-DHS_2018.cfm?flag=1.

## Declarations

### Source

Authors’ analysis based on 2018 NDHS.

## List of abbreviations

AOR: Adjusted Odds Ratio.
DHS: Demographic and Health Survey.
NDHS: Nigeria Demographic and Health Survey.
NPC: National Population Commission.
UOR: Unadjusted Odds Ratio.
